# Multidimensional Assessment of Psychiatric Adverse Events Related to Proton Pump Inhibitors: A Real‐World, Pharmacovigilance Study

**DOI:** 10.1111/cns.70436

**Published:** 2025-05-14

**Authors:** Zhi‐Qing Zhan, Jia‐Xin Li, Wei‐Gang Zhang, Shu‐Yi Huang, Xixi Fang

**Affiliations:** ^1^ Division of Gastroenterology and Hepatology, NHC Key Laboratory of Digestive Diseases, State Key Laboratory for Oncogenes and Related Genes, Shanghai Institute of Digestive Disease, School of Medicine, Renji Hospital Shanghai Jiao Tong University Shanghai China; ^2^ Department of Gastroenterology and Hepatology, West China Hospital Sichuan University Chengdu China; ^3^ The Affiliated Brain Hospital Guangzhou Medical University Guangzhou China; ^4^ Key Laboratory of Neurogenetics and Channelopathies of Guangdong Province and the Ministry of Education of China Guangzhou Medical University Guangzhou China; ^5^ Nanfang College Guangzhou China

**Keywords:** depressive symptoms, Omeprazole, proton pump inhibitors, psychiatric adverse events

## Abstract

**Introduction:**

Limited research has been conducted on the association between proton pump inhibitors (PPIs) use and psychiatric adverse events (AEs), leaving the understanding of PPIs‐related psychiatric AEs in real‐world settings unclear.

**Objectives:**

We aim to identify psychiatric AEs highly relevant to five commonly prescribed PPIs and to delve into the clinical characteristics of the population experiencing psychiatric AEs, as well as the time‐to‐onset pattern of the reported AEs.

**Methods:**

We performed disproportionality analysis to evaluate the PPI‐related psychiatric AEs risk signal using data from the FDA adverse event reporting system. Linkage disequilibrium score regression, high‐definition likelihood, and Bidirectional MR analyses were employed to evaluate genetic correlations and causality for the pairwise traits between indications for PPI therapy and three common psychiatric disorders.

**Results:**

Psychiatric AEs were reported in 12.83% of all AE reports on PPIs. Disproportionality analysis identified multiple PPI‐related psychiatric AE risk signals such as depressive disorder, bipolar disorder, and sleep disorder, with Omeprazole exhibiting the highest number of positive signals and cases (*N* = 386) and Rabeprazole the fewest (*N* = 28). Notably, we detected positive signals for suicide or self‐injury‐related AEs in three types of PPIs. Significant genetic correlations were revealed in peptic ulcer with major depressive disorder, peptic ulcer with schizophrenia, and gastroesophageal reflux disease (GERD) with major depressive disorder. Bidirectional MR analyses identified significant causal relationships between MDD and peptic ulcer, and a potential bidirectional causal association between GERD and MDD.

**Conclusions:**

PPI‐related psychiatric AEs may represent a non‐negligible portion of overall PPI‐related AEs. It is recommended to monitor and evaluate the safety of long‐term PPI use in relation to psychiatric AEs.

## Graphical abstract

Psychiatric adverse events linked to proton pump inhibitors in 12.83% of cases. Omeprazole shows the highest signal for psychiatric adverse events. Three proton pump inhibitors show signals related to suicide risks. Genetic links found between proton pump inhibitors and depression. Gastroesophageal reflux disease is linked to a higher risk of depression.

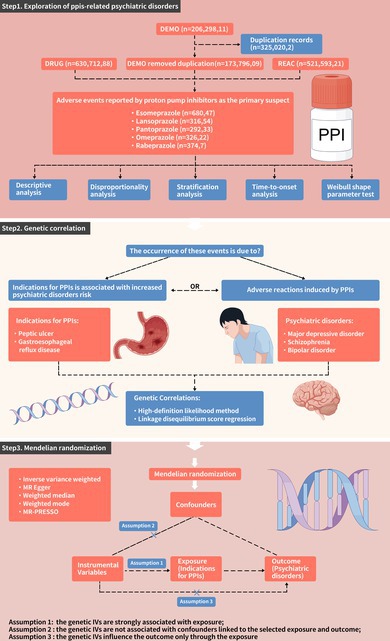

## 1 Introduction

Proton pump inhibitors (PPIs) are among the most widely prescribed medications globally [1]. A recent systematic review, which analyzed data from 28 million PPI users across 23 countries, revealed that nearly one‐quarter of adults utilize PPIs [2]. These medications are primarily utilized in the management of gastric and duodenal ulcers as well as gastroesophageal reflux disease (GERD) [1]. Chronic GERD, if left untreated, can lead to severe complications such as Barrett's esophagus, a precancerous condition that significantly increases the risk of esophageal adenocarcinoma [3]. Similarly, untreated gastric ulcers may progress to gastric adenocarcinoma, particularly in cases associated with *Helicobacter pylori* infection [4]. Thus, PPIs play a critical role in mitigating these risks by reducing gastric acid secretion, promoting ulcer healing, and preventing disease progression [1]. For a considerable period, PPIs were generally considered safe for both short‐ and long‐term use. Nevertheless, conflicting studies in recent years have raised doubts about their absolute safety, especially for long‐term users, with reports suggesting an elevated risk of anxiety and cognitive deficits [1, 5, 6]. Prior research on the correlation between PPI use and psychiatric adverse events (AEs) remains limited and has predominantly centered on depression. Laudisio et al. suggested that ceasing PPI use might be associated with a potential 14% reduction in depression cases. Conversely, no association was observed with antacids or H2 blockers [1]. A cross‐sectional study by Fong et al. similarly identified a notable link between PPI use and suicidal ideation and depression [7]. The prevalence of psychiatric AEs linked to PPIs in real‐world settings remains unclear and has rarely been explored in preapproved pivotal trials. A thorough comprehension of the psychiatric AEs of PPIs is vital to guarantee their safe clinical utilization in real‐world scenarios.

**FIGURE 1:**
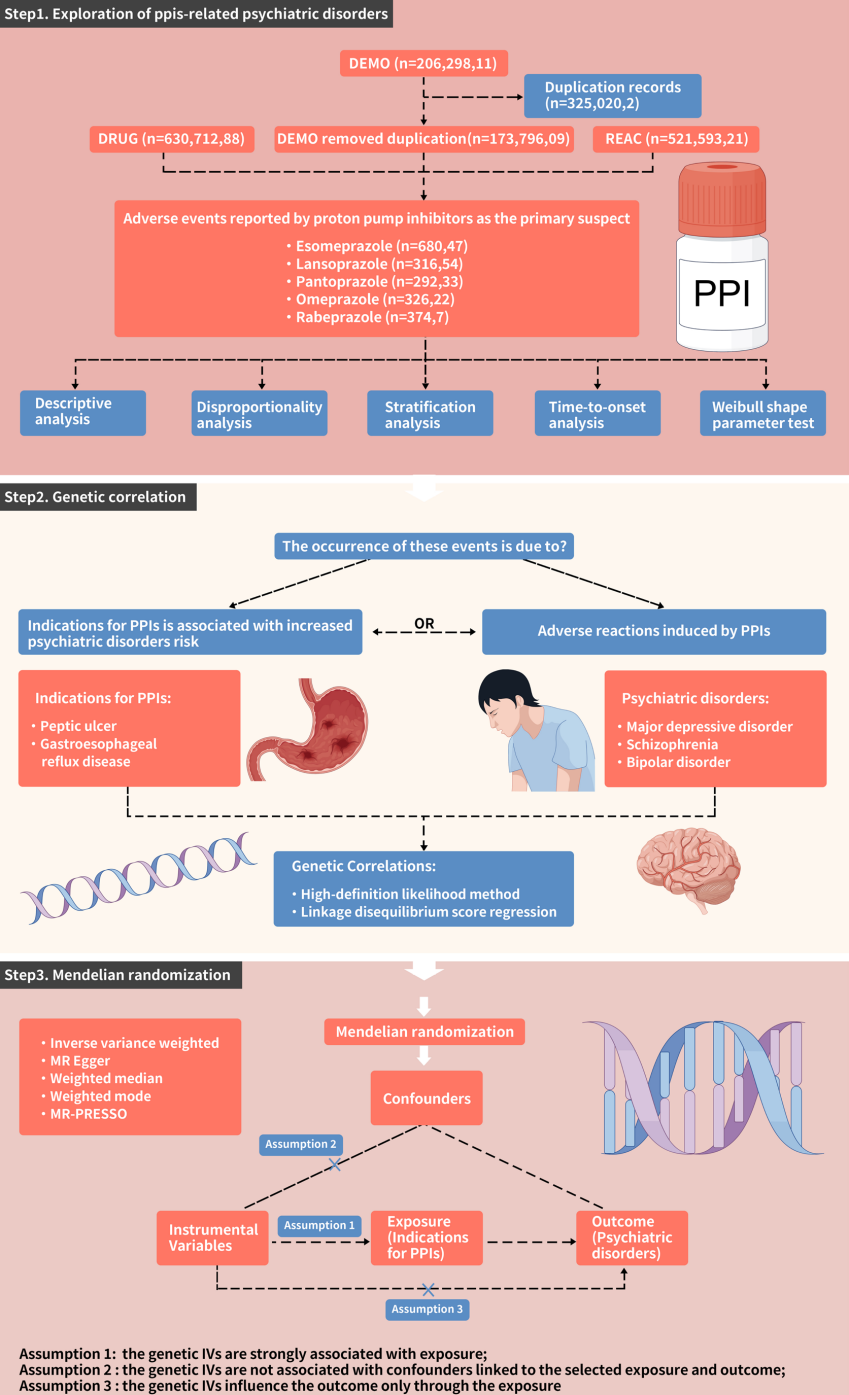
Study design. IVs, instrumental variables; PPIs, proton pump inhibitors.

Moreover, in most of the PPI users, there was no clinical evidence of underlying psychiatric disorders before the initiation of PPI therapy. Therefore, two hypotheses can be posited to explain these events: either the occurrence of psychiatric disorders is linked to the indications for PPI therapy (i.e., GERD and gastroduodenal ulcers may potentially increase the risk of subsequent psychiatric disorders), or these are AEs induced by the PPIs themselves. This remains an interesting question that requires further investigation. In this study, we first conducted a pharmacovigilance analysis to explore the association between PPI and psychiatric disorder reports by utilizing the Food and Drug Administration (FDA) adverse event reporting system (FAERS) database. Then, we utilized linkage disequilibrium score regression (LDSC) [8] and high‐definition likelihood [9] to assess genome‐wide genetic correlations for the pairwise traits between indications for PPI therapy (e.g., GERD and peptic ulcer) and three common psychiatric disorders. Finally, we conducted bidirectional Mendelian Randomization (MR) [10] to evaluate the causality between indications for PPI therapy and psychiatric disorders. Figure 1 illustrates the study workflow.

## 2 Methods

### 2.1 Data Sources of Pharmacovigilance Analysis

Managed by the FDA, FAERS is a publicly accessible post‐marketing pharmacovigilance database that aggregates data on millions of AEs worldwide for approved medicinal and biological products. FAERS facilitates the analysis of unexpected AE patterns that may be unnoticed in clinical trials as a result of participant constraints [11]. This study focuses on the commonly used PPIs: Esomeprazole, Lansoprazole, Pantoprazole, Omeprazole, and Rabeprazole. Adverse event reports associated with these PPIs were extracted from the FAERS released dashboard using both the generic and trade names as search keywords, encompassing data from the establishment of the database on January 1, 2004, to the fourth quarter of 2023. Only medications identified as primary suspect agents were included in the analysis. Adverse reactions documented in the FAERS database are categorized using preferred term (PT) codes derived from the medical dictionary for regulatory activities (MedDRA) [12]. All psychiatric adverse reaction PTs in MedDRA (version 24.1) were obtained and utilized for the subsequent analysis.

### 2.2 Statistical Analysis of Pharmacovigilance Analysis

Disproportionality analysis is a specialized data mining technique for identifying adverse drug reaction signals in large pharmacovigilance databases [13]. It allows for the comparison of observed frequency and expected frequency of selected drugs and AEs, which helps establish a statistical association between a drug and an AE [13]. In our research, we used the reporting odds ratio (ROR), a proven method for disproportionality analysis, to pinpoint potential adverse reaction signals linked to PPIs. If the lower limit of the 95% confidence interval (CI) for the ROR value exceeds 1.0, with a minimum of three reports of the target AEs (*a* ≥ 3), it suggests a potential elevated risk of the PPI triggering the target AE, thus indicating a positive signal for PPI‐related adverse reactions. Furthermore, the ROR value can be used as an index for comparing the risk of adverse reactions among PPIs, where a higher ROR value suggests a relatively higher AE risk associated with a PPI [13]. In order to delve deeper into the relationship between PPIs and psychiatric AEs, stratification analyses were carried out based on age categories (0–18, 18–65, ≥ 65 years) and gender (female and male). Bonferroni correction was employed to address the issue of multiple testing [14].

To evaluate if the occurrence of AEs would rise or decline over time, we conducted a Weibull shape parameter (WSP) test. The Weibull distribution, often employed to model reliability and lifespan data, uses the scale parameter (*α*) and shape parameter (*β*) to depict the distribution [15]. We computed the median onset time for AEs using the equation: Time‐to‐onset = event time–start time [16]. The WSP test results identify three hazard models [17]: (1) wear out failure, which implies a rising risk of AEs over time (*β* > 1, 95% CI > 1); (2) early failure, which signals a decreasing risk of AEs over time (*β* < 1, 95% CI < 1); and (3) random failure, which indicates a steady risk of AEs over time (95% CI of *β* includes 1). These tests were carried out using Minitab statistical software.

### 2.3 Genetic Correlation Analysis

Table S1 presents the GWAS data used for the genetic correlation analysis. We applied both LDSC [8] and high‐definition likelihood method [9] to assess the genome‐wide genetic correlations for six pairwise traits, relating two PPI therapy indications and three prevalent psychiatric disorders. LDSC studies the connection between test statistics and linkage disequilibrium to measure the inflation contribution from an authentic polygenic signal or bias. This approach allows for the evaluation of genetic correlation using GWAS summary statistics, unaffected by sample overlap [8]. Unlike LDSC, which only considers partial LD information, high‐definition likelihood can fully incorporate LD across the genome, greatly improving estimation accuracy [9]. The LDSC analysis was performed using the “ldscr” R package, whereas the high‐definition likelihood analysis was executed with the “HDL” R package.

### 2.4 Bidirectional MR Analyses

Bidirectional MR analysis uses genetic variants related to the exposure as instrumental variables (IVs) to identify potential causality of risk factors associated with the outcome [18]. Table S1 displays the GWAS data used for MR analysis. The choice of IVs was based on the following criteria [18]: (1) IVs should have a strong correlation with the exposures; (2) IVs should be unconnected to confounding factors; and (3) IVs should not directly correlate with the outcomes. To satisfy the first criterion, SNPs linked to each trait were selected at the genome‐wide significance threshold of *p* < 5 × 10^−8^ [18]. Only SNPs with a long physical distance (≥ 10,000 kb) and a low likelihood of linkage disequilibrium (*R* ^2^ < 0.001) were retained. In this study, we investigated the causal relationship between two indications for PPI therapy and three psychiatric disorders using five methods: inverse variance weighted (IVW), weighted median, MR‐Egger, MR‐pleiotropy residual sum and outlier (MR‐PRESSO), and weighted mode [18]. The IVW method is regarded as the most accurate and robust approach for estimating causal effects when all selected SNPs are valid IVs. MR‐Egger intercept's test was used to assess the presence of potential directional pleiotropy in the genetic variants [19]. The false discovery rate (FDR) was employed to correct for multiple testing, with FDR *q* values < 0.05 considered indicative of significance. The MR analysis was conducted using R (version 4.1.3) and the “Two‐Sample MR” package.

## 3 Results

### 3.1 Descriptive Analysis of Psychiatric AEs in PPI Patients

Figure 1 illustrated the detailed data processing. Table S2 presented the clinical characteristics of AEs associated with PPIs. A total of 20,629,811 records was extracted from the FAERS database. After the thorough elimination of duplicate entries, the final count of records amounted to 17,379,609, out of which 165,303 reports were specifically linked to AEs associated with PPIs, namely: Esomeprazole (*n* = 68,047), Lansoprazole (*n* = 31,654), Pantoprazole (*n* = 29,233), Omeprazole (*n* = 32,622), and Rabeprazole (*n* = 3747). Out of all the reports on PPIs, psychiatric AEs were reported in 12.83% of the total cases (21,211 out of 165,303). The incidence of psychiatric AEs varied among the five PPIs. The ranking of the proportion of psychiatric AEs compared to all AEs for each PPI is as follows: Omeprazole, 19.73% (6437/32,622); Esomeprazole, 12.98% (8832/68,047); Pantoprazole, 11.77% (3441/29,233); Rabeprazole, 7.77% (291/3747); and Lansoprazole, 6.98% (2210/31,654). We stratified patients into four distinct age groups (< 18, 18–65, 65–85, > 85), with the majority of PPI‐treated patients falling within the 18–65 years age group (*N* = 48,977), followed by the 65–85 years age group (*N* = 33,147). Across all PPIs, females account for a higher proportion of PPI‐related psychiatric AEs than males, particularly with Esomeprazole (female: 56.8% vs. male: 29.2%). Overall, psychiatric AEs associated with PPI treatment strategies may account for a non‐negligible proportion of their overall AEs.

### 3.2 Disproportionality Analysis for PPIs‐Related Psychiatric AEs

**FIGURE 2:**
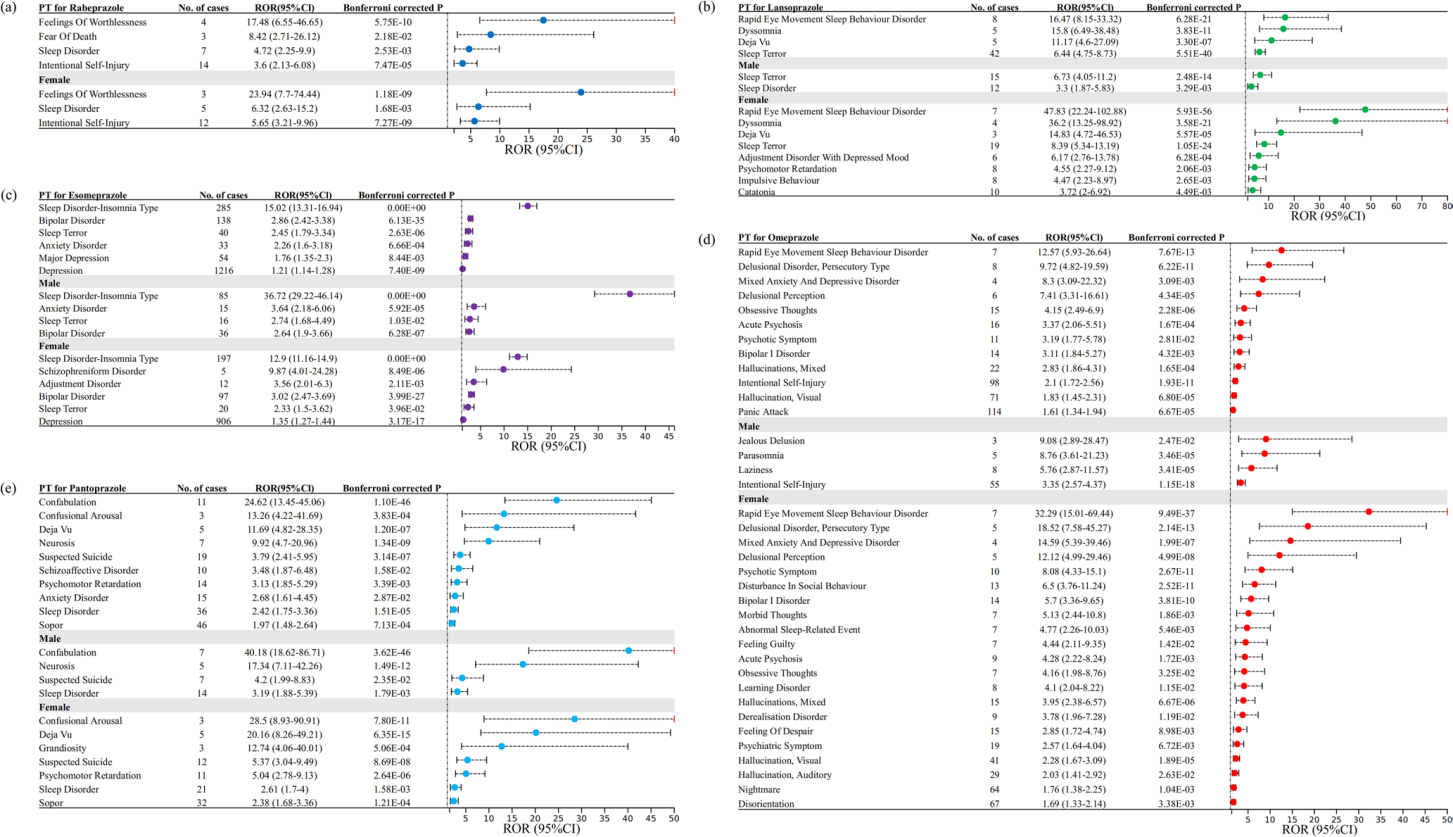
PPI‐related psychiatric AEs that still met predetermined significant thresholds after multiple testing (overall analysis and subgroup analysis by sex). (a) Rabeprazole‐related psychiatric AEs; (b) Lansoprazole‐related psychiatric AEs; (c) Esomeprazole‐related psychiatric AEs; (d) Omeprazole‐related psychiatric AEs; and (e) Pantoprazole‐related psychiatric AEs.

**FIGURE 3:**
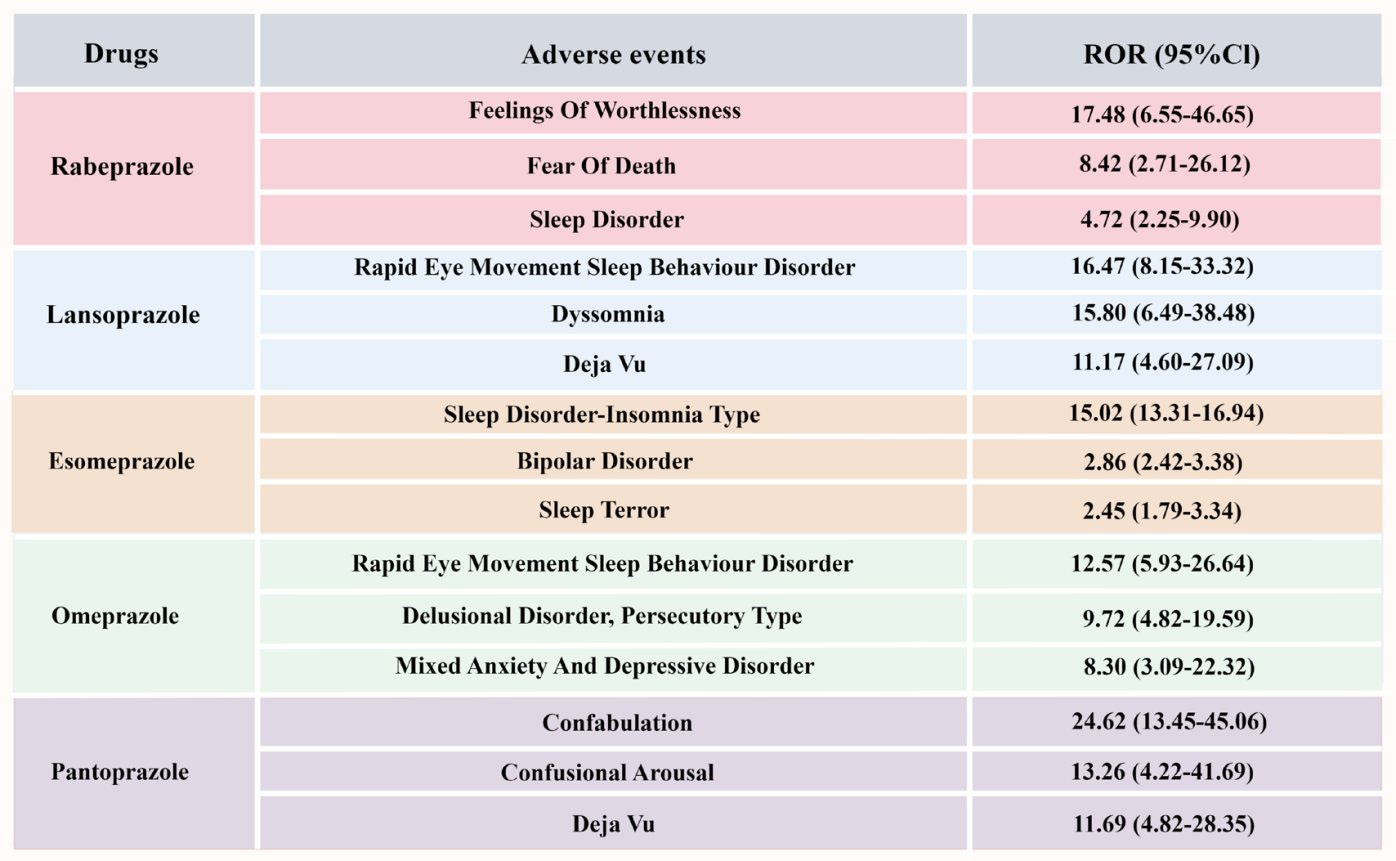
Summary of key findings in the disproportionality analysis.

PPI‐related psychiatric AEs that still met predetermined significant thresholds after multiple testing are shown in Figures 2 and 3. The psychiatric AEs common to all five types of PPIs are depicted in Figure S1. For Esomeprazole, depression (*N* = 1216, ROR [95% CI] = 1.21 [1.14–1.28]), sleep disorder–insomnia type (*N* = 285, ROR [95% CI] = 15.02 [13.31–16.94]), and bipolar disorder (*N* = 138, ROR [95% CI] = 2.86 [2.42–3.38]) were the top 3 PTs of psychiatric AEs with the highest numbers of cases. Similar positive signals were observed in the Pantoprazole‐related PTs: sleep disorder (ROR [95% CI] = 2.42 [1.75–3.36]), anxiety disorder (ROR [95% CI] = 2.68 [1.61–4.45]), and schizoaffective disorder (ROR [95% CI] = 3.48 [1.87–6.48]). For Omeprazole, rapid eye movement sleep behavior disorder (REMBD; ROR [95% CI] = 12.57 [5.93–26.64]), delusional disorder, persecutory type (ROR [95% CI] = 9.72 [4.82–19.59]), and mixed anxiety and depressive disorder (ROR [95% CI] = 8.3 [3.09–22.32]) exhibited strong positive signals. Psychiatric PTs most correlated with Lansoprazole use are mainly concentrated in sleep terror (ROR [95% CI] = 6.44 [4.75–8.73]) and REMBD (ROR [95% CI] = 16.47 [8.15–33.32]). The number of psychiatric AEs reports related to Rabeprazole is the lowest among all PPIs, with feelings of worthlessness exhibiting the most significant signal strength (ROR [95% CI] = 17.48 [6.55–46.65]) related to Rabeprazole. It should be noted that we observed significant positive signals for suicide or self‐injury related AEs in three out of five PPIs. Specifically, Pantoprazole showed a risk associated with suspected suicide (*N* = 19, ROR [95% CI] = 3.79 [2.41–5.95]). Omeprazole (*N* = 98, ROR [95% CI] = 2.1 [1.72–2.56]) and Rabeprazole (*N* = 14, ROR [95% CI] = 3.6 [2.13–6.08]) both displayed an association with intentional self‐injury.

### 3.3 Stratified Analyses Reveal Positive Signals Specific to Sex and Age Groups

**FIGURE 4:**
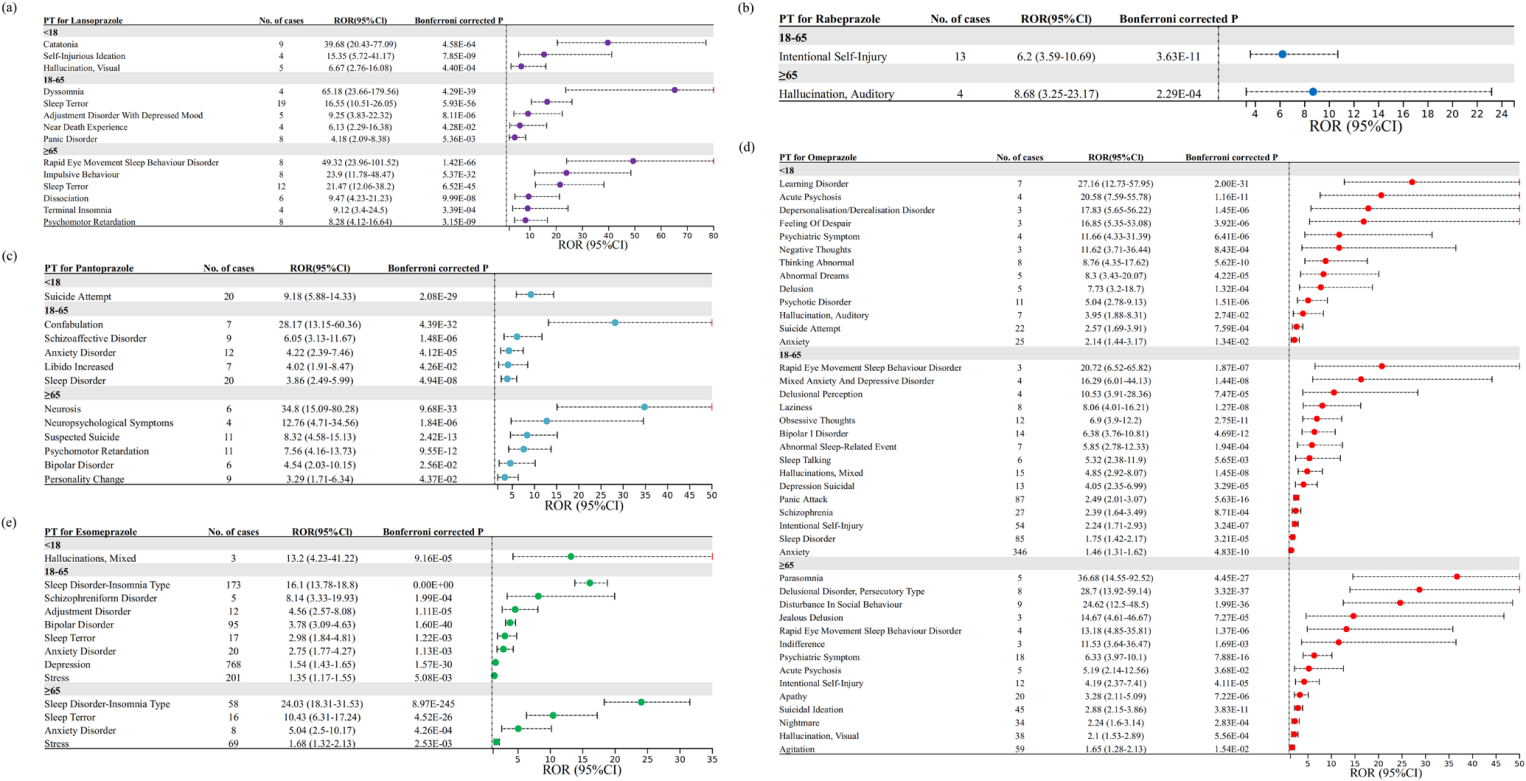
PPI‐related psychiatric AEs that still met predetermined significant thresholds after multiple testing (subgroup analysis by age). (a) Lansoprazole‐related psychiatric AEs; (b) Rabeprazole‐related psychiatric AEs; (c) Pantoprazole‐related psychiatric AEs; (d) Omeprazole‐related psychiatric AEs; and (e) Esomeprazole‐related psychiatric AEs.

The results of stratified analyses are displayed in Figures 2 and 4. It is noteworthy that we observed more cases and a stronger positive signal in females than in males, regardless of the type of PPIs. For Esomeprazole, the specific positive signal in females is depression (*N* = 906, ROR [95% CI] = 1.35 [1.27–1.44]), whereas the specific positive signal in males is anxiety disorder (*N* = 15, ROR [95% CI] = 3.64 [2.18–6.06]). Sleep disorder–insomnia type, sleep terror, and bipolar disorder are common positive signals in both males and females, but the number of cases in females is higher than in males. The gender differences in psychiatric AEs are more pronounced in Omeprazole, with disorientation (*N* = 67, ROR [95% CI] = 1.69 [1.33–2.14]), nightmare (*N* = 64, ROR [95% CI] = 1.76 [1.38–2.25]), and visual hallucination (*N* = 41, ROR [95% CI] = 2.28 [1.67–3.09]) ranking as the top three specific positive signals in females. Interestingly, no positive signals were observed in males in the analysis of Rabeprazole. Regarding AEs related to suicide or self‐injury, suspected suicide emerged as a common Pantoprazole‐related positive signal among both genders, with a higher risk observed in females (*N* = 12, ROR [95% CI] = 5.37 [3.04–9.49]) compared to males (*N* = 7, ROR [95% CI] = 4.2 [1.99–8.83]). In the case of Omeprazole, intentional self‐injury as a positive signal was only observed in males (*N* = 55, ROR [95% CI] = 3.35 [2.57–4.37]).

Stratified analyses by age revealed that the majority of PPI‐related psychiatric AEs occurred in the 18–65 age group and also identified age‐specific positive signals. Depression (*N* = 768, ROR [95% CI] = 1.54 [1.43–1.65]) and bipolar disorder (*N* = 95, ROR [95% CI] = 3.78 [3.09–4.63]) are Esomeprazole‐related psychiatric PTs specific to the 18–65 age group. For Omeprazole, anxiety was observed as a positive signal in both the < 18 years (*N* = 25, ROR [95% CI] = 2.14 [1.44–3.17]) and 18–65 years age groups (*N* = 346, ROR [95% CI] = 1.46 [1.31–1.62]), but not in the ≥ 65 years age group. Sleep disorder (*N* = 85, ROR [95% CI] = 1.75 [1.42–2.17]) and agitation (*N* = 59, ROR [95% CI] = 1.65 [1.28–2.13]) were specific Omeprazole‐related psychiatric AEs for the 18–65 and ≥ 65 years age groups, respectively. In the context of AEs related to suicide or self‐injury, we found that Omeprazole exhibited the highest number of cases, covering various age groups: suicide attempt (*N* = 22, ROR [95% CI] = 2.57 [1.69–3.91]) for individuals under 18 years old, intentional self‐injury (*N* = 54, ROR [95% CI] = 2.24 [1.71–2.93]) for individuals aged 18–65, and suicidal ideation (*N* = 45, ROR [95% CI] = 2.88 [2.15–3.86]) for individuals aged 65 and older. In the analysis for Pantoprazole, such AEs included: suicide attempt (*N* = 20, ROR [95% CI] = 9.18 [5.88–14.33]) for individuals under 18 years old and suspected suicide (*N* = 11, ROR [95% CI] = 8.32 [4.58–15.13]) for individuals aged 65 and older. For Rabeprazole, intentional self‐injury was found as a positive signal only in the age group of 18–65 (*N* = 13, ROR [95% CI] = 6.2 [3.59–10.69]).

### 3.4 Time‐To‐Onset Analysis and WSP Test

The time to PPIs‐related psychiatric AEs onset is depicted in Figure S2, and the results of the WSP test are presented in Table 1. The median onset times for depression induced by the five PPIs are as follows: 26.5 days for Pantoprazole, 83 days for Lansoprazole, 96 days for Omeprazole, 168 days for Rabeprazole, and 716 days for Esomeprazole. The three PPIs with the earliest median onset times for inducing anxiety are Esomeprazole at 91 days, Lansoprazole at 123 days, and Rabeprazole at 132 days. Esomeprazole has the earliest median onset time for inducing sleep disorders at 19 days, whereas Lansoprazole has the latest at 214 days. In the evaluation of the WSP analysis, the shape parameter (*β*) for all 5 types of PPIs was calculated to be within the range of 0–1, suggesting that the incidence of AEs was considered to decrease over time, indicating an early failure type.

**TABLE 1:** Results of time‐to‐onset analysis and Weibull shape parameter test.

Drug	Time‐to‐onset (days)	Weibull distribution		Failure type
		Scale parameter	Shape parameter	
	Median (IQR)	*α* (95% CI)	*β* (95% CI)	
Esomeprazole	23.5 (3–380)	142.46 (110.75–174.16)	0.39 (0.37–0.42)	Early failure
Lansoprazole	31 (4–214)	129.69 (91.09–168.29)	0.38 (0.35–0.41)	Early failure
Pantoprazole	9 (2–34)	41.88 (31.67–52.09)	0.39 (0.37–0.42)	Early failure
Omeprazole	9 (4–96)	69.05 (57.35–80.74)	0.39 (0.38–0.41)	Early failure
Rabeprazole	51 (4–168)	78.95 (40.82–117.08)	0.53 (0.43–0.63)	Early failure

Abbreviations: CI, confidence interval; IQR, interquartile range.

### 3.5 Results of Genetic Correlations and Bidirectional MR Analyses

The results of genetic correlations between indications for PPIs and three psychiatric disorders are presented in Table 2. After adjusting for multiple testing, three pairs of traits exhibited simultaneous significant genome‐wide genetic correlations in both the high‐definition likelihood method and LDSC method, including: peptic ulcer with major depressive disorder (MDD), peptic ulcer with schizophrenia, and GERD with MDD (Table 2). The results of bidirectional MR between indications for PPIs and three psychiatric disorders are presented in Table S3. Bidirectional MR analysis revealed that genetically predicted MDD causally increased the risk of peptic ulcer (OR = 1.27, 95% CI = 1.11–1.45, FDR = 0.002). Additionally, a potential bidirectional causal relationship was identified between GERD and MDD, with GERD to MDD (OR = 1.18, 95% CI = 1.06–1.31, FDR = 0.008) and MDD to GERD (OR = 1.37, 95% CI = 1.27–1.48, FDR = 3.87 × 10^−15^). No significant causal relationship was found in the other pairs. For all the pairs examined, the MR‐Egger intercept tests yielded FDR > 0.05, indicating no significant evidence of pleiotropy (Table S3).

**TABLE 2:** Results of genetic correlations analysis.

Diseases pairs	Genetic correlations			
	High‐definition likelihood method		LDSC method	
	rg (SE)	FDR	rg (SE)	FDR
Peptic ulcer and MDD	0.4818 (0.0422)	1.52 × 10^−29^	0.4438 (0.0437)	1.33 × 10^−23^
Peptic ulcer and schizophrenia	0.1162 (0.0283)	6.42 × 10^−5^	0.1306 (0.0402)	1.92 × 10^−3^
Peptic ulcer and bipolar disorder	0.0996 (0.0338)	4.32 × 10^−3^	0.0691 (0.0437)	0.152
GERD and MDD	0.4693 (0.0258)	3.23 × 10^−73^	0.4596 (0.0267)	2.80 × 10^−65^
GERD and schizophrenia	0.0462 (0.0185)	0.014	0.0313 (0.0273)	0.281
GERD and bipolar disorder	0.0385 (0.0224)	0.086	0.0332 (0.0308)	0.281

Abbreviations: GERD, gastroesophageal reflux disease; LDSC, linkage disequilibrium score regression; MDD, major depressive disorder.

## 4 Discussion

Previous studies on PPIs have predominantly focused on their mechanism of action and clinical trials, with limited emphasis on recent real‐world research concerning psychiatric AEs in PPI patients. This study is the first to identify psychiatric AEs highly relevant to PPI treatment through disproportionality analysis and to explore the clinical characteristics, including age and gender specificity, as well as onset time of the reported AEs. We further revealed genetic correlations between peptic ulcer with MDD, peptic ulcer with schizophrenia, and GERD with MDD. Bidirectional MR analyses were conducted among six pairwise traits, and the results revealed significant causal relationships between MDD and peptic ulcer. Additionally, a potential bidirectional causal association was identified between GERD and MDD. However, no significant causal associations were detected in the remaining pairs.

**FIGURE 5:**
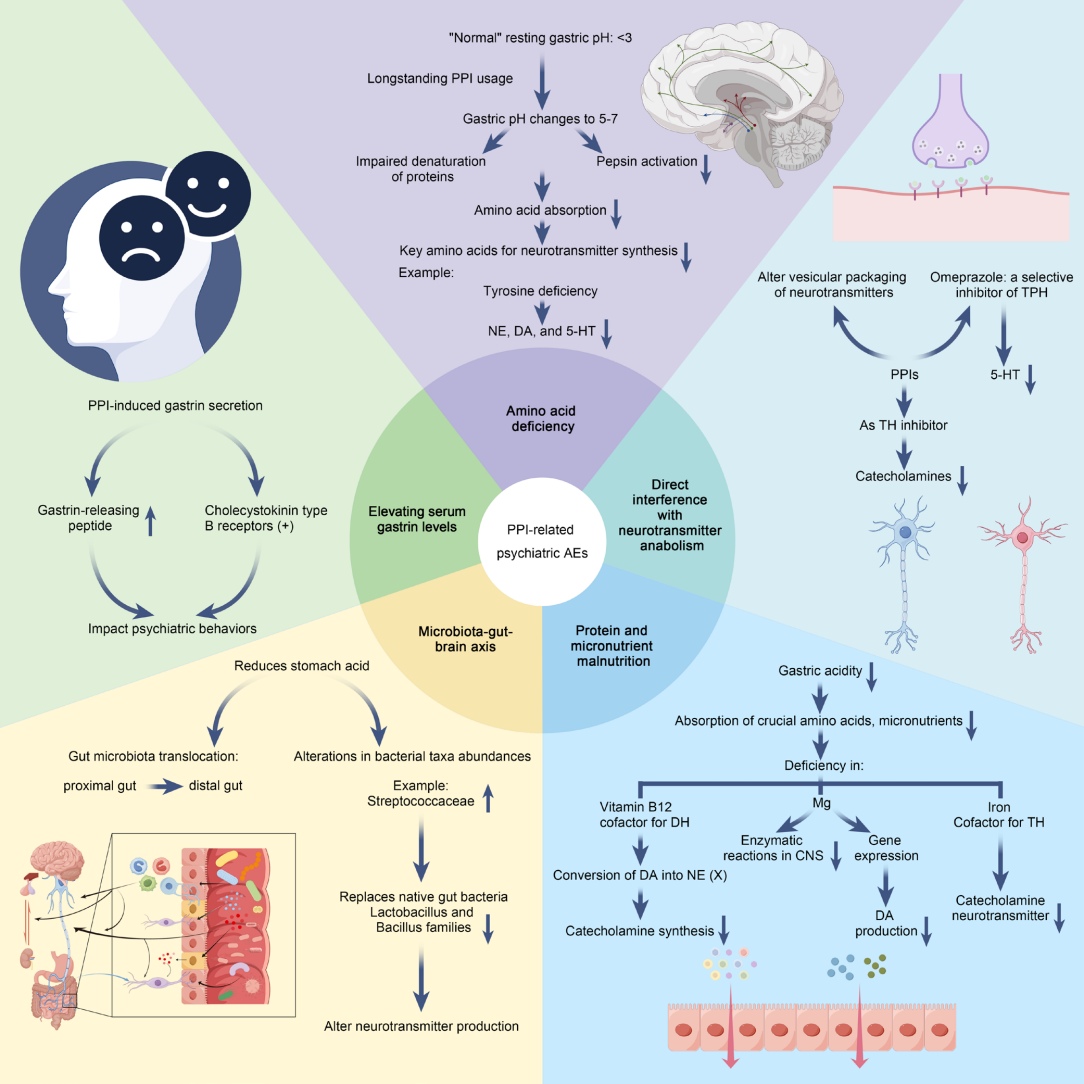
The potential mechanisms by which chronic use of PPIs induces psychiatric disorders. 5‐HT, 5‐hydroxytryptamine (serotonin); CNS, central nervous system; DA, dopamine; DH, dopamine hydroxylase; NE, norepinephrine; TH, tyrosine hydroxylase; TPH, tryptophan hydroxylase.

Previous evidence regarding PPI‐related psychiatric AEs has been limited and primarily focused on depression and anxiety. A dose‐effect relationship has been identified in the association of PPI use with mood alterations [1]. Higher PPI exposure was linked to an elevated risk of subsequent major depression [20]. Similarly, a study by Wang et al. revealed that the initiation of PPI use in children, compared to nonuse, was significantly associated with an approximately 2.6‐fold increased risk of depression and anxiety [21]. Furthermore, the use of Rabeprazole has been implicated in inducing anxiety and panic attacks [22]. Our study corroborates these findings and further unveils that PPI use may be associated with a broader spectrum of psychiatric disorders, such as bipolar disorder, schizophreniform disorder, and schizoaffective disorder. Chronic use of PPIs may induce psychiatric disorders through several mechanisms (Figure 5) [23]: (1) Elevating serum gastrin levels. Evidence has demonstrated that gastrin‐releasing peptide, whose release is mediated by PPI‐induced secretion of gastrin, plays a role in regulating aspects of behavior that could be modified in conditions including anxiety and depression [24, 25]. Additionally, hypergastrinemia activates the central nervous system's cholecystokinin type B receptors, impacting anxiety, feeding, and locomotion [25, 26]. (2) Direct interference with neurotransmitter anabolism. In the cerebral context, PPIs influence neurotransmission through alterations in the vesicular packaging of neurotransmitters such as acetylcholine, GABA, and glutamate, among others [27]. In in vivo mouse models, Omeprazole was demonstrated to be a selective inhibitor of tryptophan hydroxylase, a key enzyme in the synthesis of serotonin (5‐HT) [28]. Chronic use of Omeprazole has been associated with decreased serotonin neurotransmission in the raphe hippocampus and reduced dopamine metabolism, potentially leading to anxiety and depression [24]. (3) Impacting neurotransmission through protein and micronutrient malnutrition. Numerous studies suggest that PPIs alter gastric acidity, leading to chronic malabsorption of proteins and micronutrients crucial for neurotransmitter synthesis [29, 30, 31]. Vitamin B12, a key enzymatic cofactor in catecholamine synthesis, is often deficient due to PPI use [30, 32]. This deficiency can limit catecholamine synthesis and has been associated with a twofold increase in severe depression risk [33]. Meta‐analyses suggest that long‐term PPI use can lead to hypomagnesemia [34]. Magnesium is crucial for over 300 human enzymatic reactions and modulates dopamine production [35]. A meta‐analysis found that hypomagnesemia is associated with a 1.3‐fold increased depression risk [36]. Observations of PPI‐associated iron‐deficiency anemia [37], coupled with iron's role as a cofactor for tyrosine hydroxylase in catecholamine neurotransmitter production [38], suggest that long‐term PPI use could impact psychiatric disorders by limiting iron absorption and consequently catecholamine neurotransmitter production [23]. (4) Microbiota‐gut‐brain axis (MGBA). Long‐term PPI use reduces stomach acid, prompting a gut environment shift that facilitates the translocation of proximal gut bacterial populations to the distal region and alters certain bacterial taxa abundances [39]. PPI‐induced overgrowth of *Streptococcaceae* replaces native gut bacteria, such as *Lactobacillus* and *Bacillus* families, affecting neurotransmitter production and potentially causing anxiety and depression [40]. In addition, similarities can be observed in different bacterial taxa levels while comparing depressed people's gut microbiota with the PPIs‐induced dysbiosis [41].

Another significant finding of this study is the association between PPI use and sleep disorder, particularly REMBD, as well as AEs related to suicide or self‐injury, irrespective of the type of PPI. Chronic REM sleep deprivation can lead to mental issues like learning and memory deficits and psychiatric disorders such as anxiety and depression [42]. Lansoprazole has been demonstrated to inhibit neuronal activity in the locus coeruleus, affecting REM sleep in rats [42]. Similarly, PPIs have been reported to induce REMBD in humans, which aligns with our findings [43]. Fong et al. found that PPI users had a 2.34‐fold higher risk of suicidal ideation than nonusers, particularly among those aged 40–49 [7]. Our study aligns with this, noting increased suicide or self‐injury related events in all ages among Omeprazole users, with the highest case count and signal strength found in the 18–65 age bracket. Further longitudinal studies are needed to fully understand these associations and potential implications, such as monitoring depression severity or associated risks during PPI use. Notably, our study also revealed differences among various PPIs in inducing psychiatric AEs. We found that Omeprazole and Esomeprazole were associated with a higher risk of psychiatric AEs including depression, anxiety, and bipolar disorder. These two PPIs were demonstrated to have greater irreversibility in inhibiting CYP2C19 compared to other PPIs [20]. In addition, different PPIs may lead to distinct psychiatric AEs due to their unique pharmacokinetic and pharmacodynamic profiles. For example, variations in absorption, metabolism, and off‐target effects can influence central nervous system (CNS) exposure and subsequent psychiatric symptoms [44]. Further research is needed to discern whether their distinct pharmacokinetic and metabolic profiles contributed to these findings. Additionally, our findings demonstrate age and gender specificity in PPI‐related psychiatric AEs. Previous research has shown that the amount of gastric acid output declines with aging [45]. As discussed earlier, chronic PPI use may contribute to the onset of psychiatric disorders by disrupting amino acid metabolism and neurotransmitter anabolism through various mechanisms. Consequently, the disparity in gastric acidity between PPI users and nonusers may differ with age, thus resulting in variations in psychiatric AEs across different age groups. Sex and gender‐based differences in drug metabolism exist at multiple levels, potentially resulting from the genomic and non‐genomic actions of sex hormones [46]. Furthermore, numerous studies have established that men and women exhibit different susceptibilities to psychiatric disorders [47]. For instance, women are more prone to anxiety disorders, depression, and late‐onset schizophrenia, whereas men are more likely to experience early‐onset schizophrenia [47]. Future research should focus on elucidating these mechanisms and developing personalized treatment strategies to minimize psychiatric AEs.

Several limitations of our research should be acknowledged. First, FAERS is subject to several biases, including selection bias, underreporting, reporting bias, and inaccuracies. These limitations may influence the interpretation of our findings. Therefore, our results should be interpreted with caution and require further validation through prospective studies. Second, FAERS cannot fully account for confounding factors. To address this, we conducted stratified analyses by sex and age, revealing subgroup‐specific positive signals. This approach helped identify differences in adverse event incidence and reporting across subgroups, partially mitigating confounding effects. However, unmeasured factors, such as comorbidities, concurrent medications, and detailed PPI use information may still affect the observed associations between PPI use and psychiatric disorders. Future research should use controlled designs, such as prospective cohorts or randomized trials, to better establish causal relationships. Third, inadequate control for baseline psychiatric conditions and lack of independent validation data limit our findings. A larger prospective study with comprehensive assessments and follow‐up is needed for robust evidence. At last, the GWAS data used in our genetic correlation analysis lack information on PPI use timing and psychiatric symptom onset, restricting conclusions about temporal sequences. Future studies should prioritize collecting detailed temporal data through longitudinal cohorts or electronic health records to better understand the dynamics between PPI use and psychiatric outcomes.

### 4.1 Suggestions for Future Research Directions and Clinical Practice

Our findings may offer the following insights for future research directions and clinical practice: (i) Future investigations necessitate a comprehensive exploration of potential associations between PPI use and psychiatric disorders. This includes a more profound analysis of the aforementioned biochemical pathways, enhanced comprehension of patient‐specific risk factors, a more refined pharmacogenomic understanding of the drugs, standardization of serum monitoring for relevant biomarkers (e.g., neurotransmitter precursors or inflammatory cytokines), and the execution of long‐term cohort studies focused on PPI therapy. These areas of research are critical for furthering our understanding and potentially guiding therapeutic decisions; (ii) For patients with chronic gastrointestinal diseases where conventional treatment, typically involving PPIs, yields poor efficacy and there are suspicions of underlying psychological factors, the decision to use PPIs should be based on individualized considerations. Collaborative efforts between gastroenterologists and psychiatric specialists may be necessary to devise the optimal treatment plan, ensuring both safety and efficacy; (iii) For patients requiring long‐term PPI therapy, regular monitoring and follow‐ups, such as mood assessments, are advisable for dosage adjustments and treatment optimization; (iv) Should these findings be substantiated, the coadministration of drugs or supplements aimed at augmenting key trace elements and serotonin neurotransmission could prove beneficial.

## 5 Conclusions

In conclusion, our study unveiled various psychiatric AEs related to PPIs through a disproportionality analysis and also probed the genetic correlations and causality between PPI indications and common psychiatric disorders. These findings underscore the necessity for heightened awareness and more rigorous prescription guidelines concerning PPI therapeutic use. Should these findings be substantiated, the coadministration of drugs or supplements aimed at augmenting key trace elements and serotonin neurotransmission could prove beneficial.

## Author Contributions

**Zhi‐Qing Zhan:** conceptualization: equal; methodology: equal; formal analysis: equal; data curation: equal; and writing – original draft: leading. **Jia‐Xin Li:** data curation: supporting; and writing – original draft: supporting. **Wei‐Gang Zhang:** data curation: supporting. **Shu‐Yi Huang:** funding acquisition: leading; and writing – review and editing: supporting. **Xixi Fang:** funding acquisition: leading; and writing – review and editing: supporting.

## Ethics Statement

The authors have nothing to report.

## Conflicts of Interest

The authors declare no conflicts of interest.

## Supporting information


Figure S1.

Figure S2.

Table S1.
Table S2.
Table S3.

## Data Availability

Data can be obtained upon a reasonable request to zhi-qingzhan@sjtu.edu.cn.
