# Supplementary figures and images for: Multidimensional Assessment of Psychiatric Adverse Events Related to Proton Pump Inhibitors: A Real‐World, Pharmacovigilance Study
Source: CNS Neurosci Ther. 2025 May 14;31(5):e70436. doi: 10.1111/cns.70436 (PMC12076120; doi:10.1111/cns.70436)

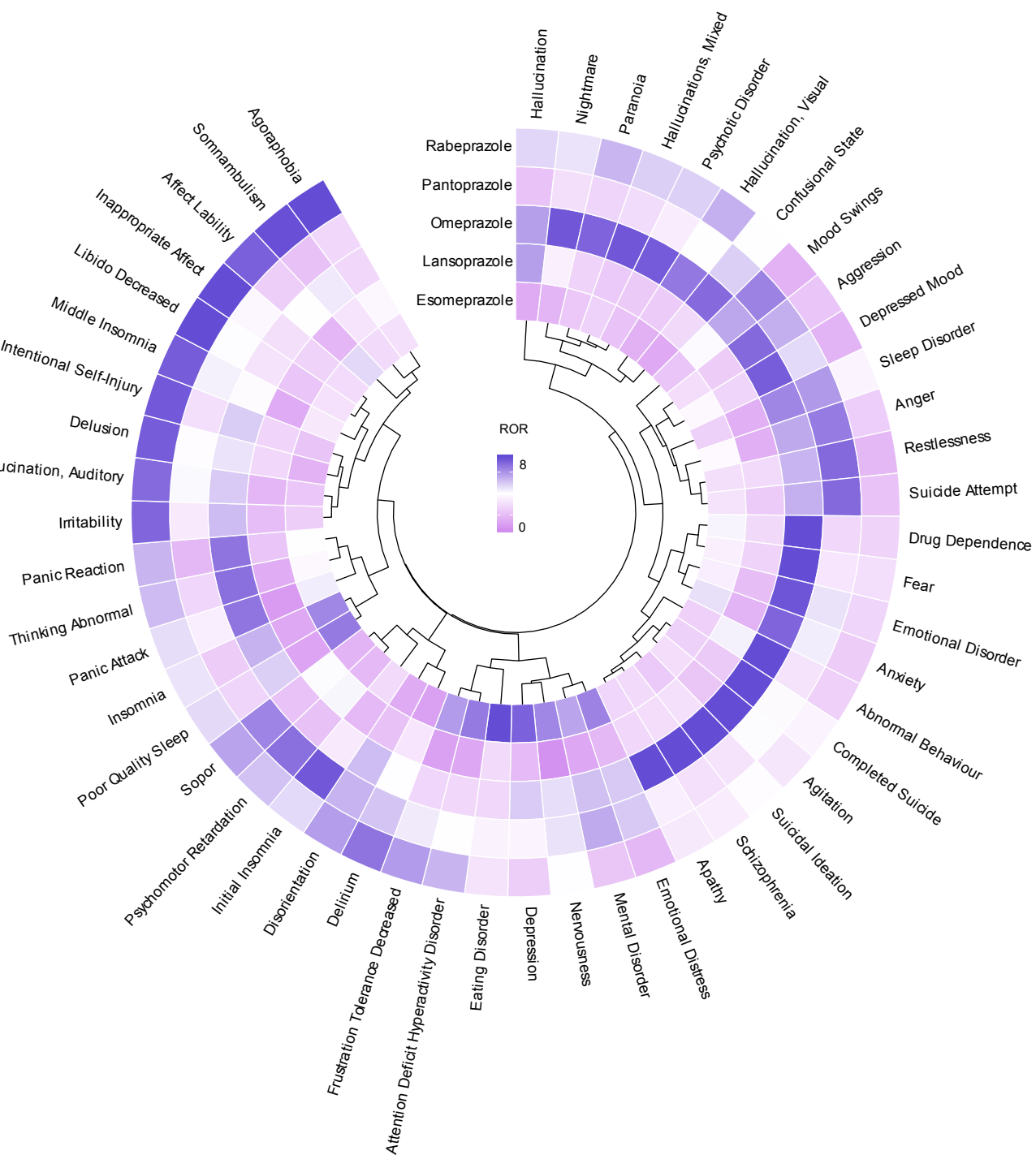

Supplement: Supplementary file 1 — Figure S1. [file CNS-31-e70436-s003.pdf]

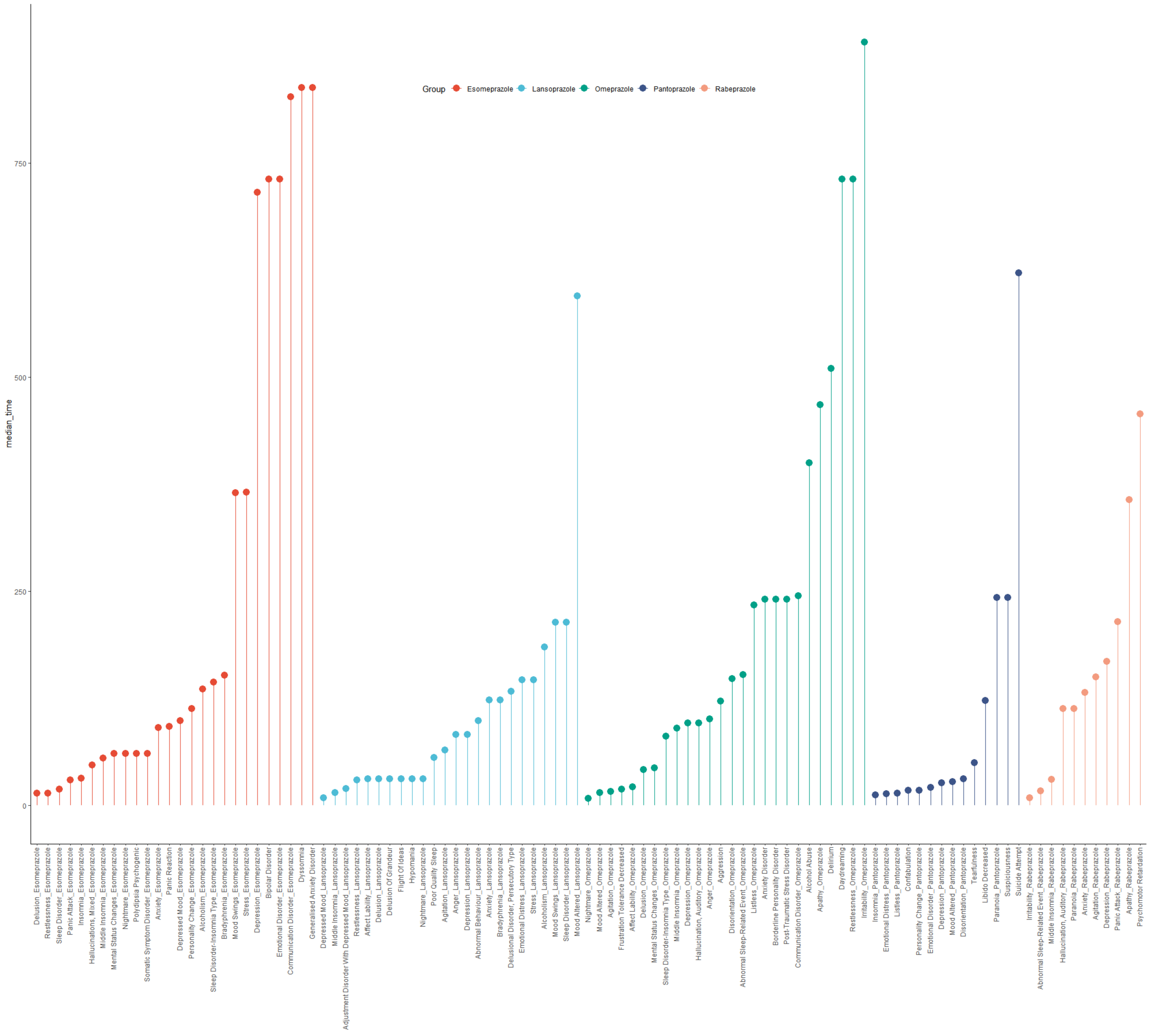

Supplement: Supplementary file 2 — Figure S2. [file CNS-31-e70436-s001.pdf]
